# Mental health and the jilted generation: Using age-period-cohort analysis to assess differential trends in young people's mental health following the Great Recession and austerity in England

**DOI:** 10.1016/j.socscimed.2018.08.034

**Published:** 2018-10

**Authors:** Rachel M. Thomson, Srinivasa Vittal Katikireddi

**Affiliations:** a MRC/CSO Social & Public Health Sciences Unit, University of Glasgow, UK; b Public Health Department, NHS Ayrshire & Arran, UK

**Keywords:** UK, Mental health, Health inequalities, Social epidemiology, Social policy, Austerity

## Abstract

Those born in the United Kingdom post-1979 have been described as a ‘jilted generation’, materially disadvantaged by economic and social policy; however, it is unclear whether this resulted in their experiencing poorer mental health than previous cohorts. Following the 2008 recession, UK austerity reforms associated with worsening mental health also disproportionately impacted those of younger working-age. This study aimed to identify any historic cohort changes in population mental health, and whether austerity widened generational inequalities. Repeat cross-sectional data from the Health Survey for England (1991–2014) were used to calculate prevalence of psychopathology for those of younger and older working-age (16–30 and 31–64 years) and retirement-age (65+ years), measured by General Health Questionnaire-12 (GHQ) score ≥ 4 (caseness). Descriptive age-period-cohort analysis was performed for 15-year birth cohorts, including the jilted generation (born 1976-90). Logistic regression tested differences in outcome between groups.

Age-specific GHQ caseness between successive birth cohorts did not significantly change for men, and significantly improved between 2.8% (95% CI 0.1%–5.5%) and 4.4% (95% CI 2.2%–6.7%) for women. Secondary analysis adjusting for education partially explained this improvement. Following the recession, GHQ caseness worsened in men of younger and older working-age by 3.7% (95% CI 1.2%–6.2%) and 3.5% (95% CI 2.1%–5.0%) respectively before returning to baseline during austerity. All women experienced non-significant increases post-recession, but trends diverged during austerity with caseness worsening by 2.3% (95% CI 1.0%–3.6%) for older working-age women versus 3.7% (95% CI 1.3%–6.2%) for younger working-age women. Those of retirement-age experienced little change throughout. In summary, mental health has historically improved between successive cohorts, including for the jilted generation. However, the 2008 recession and subsequent austerity could be most impacting those of younger working-age, particularly women, to create a new cohort effect. Policymakers should consider the differential impact economic and social policy may have across society by age.

## Highlights

• Mental health in England has consistently improved from generation to generation.
• These generational improvements have been most marked for women.
• Following the onset of austerity young women experienced worsening mental health.
• Mental health of over 65s was relatively protected during recession and austerity.
• Austerity policies have potential to widen generational inequality in mental health.

## 1 Introduction

Recessions, and the political decisions which follow them, can have significant short- and long-term health and social consequences which potentially make them of great public health importance (Stuckler et al., 2009). For mental health specifically, largely negative consequences have been observed in the aftermath of recessions (Frasquilho et al., 2016), particularly male suicides associated with the immediate spike in unemployment which often follows (Barr et al., 2012). Female mental health appears less acutely affected, for reasons which are unclear (Katikireddi et al., 2012). Such health effects are often unevenly distributed across society, with those in disadvantaged groups more likely to be heavily impacted by unemployment, potentially widening existing health inequalities (Bartoll et al., 2015; Ruckert and Labonte, 2014). There is also growing evidence that the pursuit of austerity policies in the aftermath of economic crises such as the global recession in 2008 (commonly referred to as the Great Recession) may worsen health outcomes and prolong the period of economic recovery (De Vogli, 2014; Stuckler and Basu, 2013).

Our recent work has demonstrated that, following the onset of strict austerity policies in the United Kingdom in response to the Great Recession, there was a widening of gender inequalities in poor mental health due to a marked worsening in mental health among women (Thomson et al., 2018). Others offer conflicting opinions on whether socioeconomic inequalities in poor mental health may have narrowed or widened during the same period (Barr et al., 2015; Reibling et al., 2017). However, despite some evidence that the age patterning of suicide mortality associated with the Great Recession may not be quite as would be expected, with the most marked rises occurring in younger rather than older men (Chang et al., 2013), there is little in the literature further considering whether the mental health of particular age cohorts has been disproportionately affected by either the recession or austerity.

The existence of a ‘jilted generation’ in the UK, including all those born after 1979, has previously been hypothesised to be the result of broad societal changes which occurred following this point with the rise of free market capitalism and individualism (Howker, 2013). This is thought to have led to a phenomenon where young adults are now materially disadvantaged compared with previous generations (BBC News, 2016), particularly in relation to housing (The Office for National Statistics, 2010), employment prospects (The Work Foundation, 2013), and inheritance of extremely high and unsustainable national debt (Hagist et al., 2009).

There is little further consideration found in the literature explicitly considering whether health outcomes may be similarly affected for those in this cohort. It is widely acknowledged these social determinants of health such as income, housing and employment can be thought of as the ‘causes of causes’ for a broad range of poor health outcomes (Braveman and Gottlieb, 2014), and therefore it could be hypothesised that a similar phenomenon characterised by deteriorating mental health in this group may be observed: ‘the jilted generation hypothesis’. However, health is also influenced by other factors which may mitigate any potential influence of these materials disadvantages for this cohort. Improvements in access to education and improving healthcare technologies are likely to confer advantage on this age group relative to previous generations which may balance any material loss (Hahn and Truman, 2015), particularly with the UK described as ‘leading the world’ in terms of equity of access to health services (Dayan et al., 2018).

While not discussed in these terms, there is some evidence that the jilted generation hypothesis may extend to mental health. Time-trend analysis by Chang et al. found that, in contrast with previous recessions where those over 65 years and middle-aged men were found to experience the sharpest rise in suicide rates (Gavrilova et al., 2000; Chang et al., 2009), across Europe in the year following the Great Recession the highest rise was actually among men aged 15–24 years, which they postulated may be secondary to the fact that rises in unemployment were steepest in this age group (Chang et al., 2013). Work by Coope et al. also showed that in the UK suicide rates had actually been increasing in 16–34 year old men in the period *prior* to the 2008 recession before any rises in unemployment (Coope et al., 2014). Based on this evidence, it may be possible that this post-1979 cohort was both more likely to experience poor mental health prior to the recession, and particularly vulnerable to its effects, which could be explored using age-period-cohort analysis. Of note, both authors explore only the immediate post-recession period when male mental health may be more influenced by macroeconomic factors than female mental health for reasons that are unclear (Frasquilho et al., 2016; Katikireddi et al., 2012), in contrast with the period following economic policy response where austerity policies in the UK may have had more influence on female mental health (Thomson et al., 2018).

Briefly, age-period-cohort analysis centres on trying to tease out the different impacts of each of these influences on health: the impact of *age* across an individual's life course; the impact of living through a specific *time period* where the health of all was affected by some global change in circumstances; and the separate effect of being born into a specific *birth cohort* with shared experiences causing this group to be intrinsically different from other cohorts (Suzuki, 2012). There is little recent research aiming to untangle age-period-cohort effects in relation to mental health in the UK population, and that which exists is inconclusive. Work by Bell et al. using data from the British Household Panel Survey found that more recent cohorts have poorer mental health, potentially supporting the jilted generation hypothesis (Bell, 2014). However, Spiers et al. using a similar approach with data from the Adult Psychiatric Morbidity Study found no evidence of significant cohort effects in poor mental health (Spiers et al., 2011), and in a separate study using the Health Survey for England Rice et al. found the highest prevalence of diagnosed mental illness in the ‘baby boomer’ cohort (though did not consider those of younger working age) (Rice et al., 2010). Our study aims to add clarity to these conflicting findings using a more descriptive approach to age-period-cohort analysis, overcoming some of the statistical limitations of these studies outlined below.

We aimed firstly to examine long-term trends in mental health in England to determine whether there had been a historic decline in mental health for younger birth cohorts (as per the jilted generation hypothesis), and secondly whether the recession and subsequent austerity policies may have had a differential impact across birth cohorts to create generational inequalities in poor mental health.

## 2 Methods

### 2.1 Study design

We used repeat cross-sectional data from the Health Survey for England (HSE), a multi-stage stratified random sample designed to be nationally and regionally representative, spanning 1991 to 2014. Details of the HSE have been published elsewhere (Mindell et al., 2012). Response levels have fallen over time but plateaued recently, remaining reasonably high at 62% in 2014 compared with 68% in 2006 (NatCen Social Research, 1991–2014). Weights for non-response were available from 2003. Relevant data were available for all years except 1996, 2007, 2011 and 2013 when the outcome measure was not administered.

The HSE has run for a considerable time using standardised methods with frequent data collection, allowing consideration of long-term trends. Cross-sectional rather than longitudinal data were used to allow inclusion of birth cohorts who only reached the age of 16 years during the study period, and so would not have been eligible for initial recruitment to longitudinal cohort studies of adults. This approach also avoided residual confounding that could occur using panel data which include whole households for age-period-cohort analysis, as children in included households who are subsequently followed up as adults are likely to share many genetic and environmental influences with others in their household.

### 2.2 Population

The HSE general population samples were used, and all participants over the age of 16 years were eligible for inclusion. Due to the expected small sample size following stratification by birth cohort, datasets were pooled into two year groupings to stabilise trends.

### 2.3 Exposure measurement

The UK economy did not enter recession until the last quarter of 2008 (defined by two successive quarters of negative growth in GDP) (Macrotrends, ; aThe Office for National Statistics, ), and while austerity policies were announced in mid-2010 (Reeves et al., 2013) it is unlikely that potential health consequences would have fully manifested within this year due to the time taken to achieve full implementation. To avoid misclassification of individuals we therefore defined in advance all pooled two year periods up to and including 2008 ‘pre-recession’, the period 2009/10 the ‘recession period’, and 2012/14 the ‘austerity period’.

For secondary analysis considering the potential explanatory role of educational expansion, highest educational attainment was available for all included years except 1995, coded into four categories: degree-level or equivalent, A-level or equivalent, GCSE or equivalent, and no formal qualifications.

### 2.4 Outcome measurement

Poor mental health was assessed using the General Health Questionnaire-12 (GHQ-12), a validated screening tool for common mental health problems used widely in epidemiological research, which scores self-reported symptoms of anxiety and depression (Goldberg et al., 1997). The GHQ-12 formed part of the core questions in each sweep of the HSE except 1996 and 2007, though from 2010 has only been included every second year. A GHQ-12 score of four or greater indicates a strong likelihood of a common mental disorder (Goldberg and Williams, 1988), and therefore defined a ‘case’.

### 2.5 Data analysis

The literature relating to the preferred methodology for age-period-cohort analysis is vast, and centres on the problem of identification: as there exists an exact mathematical linear dependency between the three variables (age = period – cohort), creating statistical models to determine the exact effects of one variable while controlling for the effects of the others is extremely challenging (Keyes et al., 2010). While there have been multiple complex statistical methods developed over the last 30 years as a potential way of addressing this, they all have significant limitations and/or require strong assumptions on the part of the researcher based on intuition (Bell and Jones, 2014); in fact, it has been suggested that it may well be a ‘logically impossible’ problem (Glenn, 2005). As such, a more traditional descriptive approach to considering age-period-cohort effects was chosen which, though not able to make a quantitative assessment of the differences across the whole time period considered, gives a qualitative visual impression of these effects supported by simple statistical tests of difference.

Firstly, to provisionally examine both age and period effects, age-specific prevalence estimates of GHQ caseness (with 95% confidence intervals) were calculated and plotted over time for each pooled two-year period. Three age bands were selected: 16–30 years (younger working-age), 31–64 years (older working-age) and 65+ years (retired), based on the observation that UK employment rates fell most sharply for those of younger working-age following the onset of the recession and so the mental health of these groups may have been differentially impacted (bThe Office for National Statistics, ).

Secondly, in order to examine cohort effects, five 15-year birth cohorts were created within the pooled datasets. From pooled datasets 1991/92 to 2004/05 only four birth cohorts were included: those born in 1916-30 (WWI cohort), 1931-45 (WWII cohort), 1946-60 (baby boomer cohort) and 1961-75 (generation X cohort). From data collection years 2006/08 onwards, the WWI cohort was removed due to decreasing sample size, and a new cohort of those born 1976-90 was generated: this cohort was taken to represent the ‘jilted generation’.

Prevalence estimates of GHQ caseness with 95% confidence intervals were then calculated within each pooled dataset for all birth cohorts. These were plotted in three ways:

• Over the life course, to see how age effects may vary between cohorts
• Over time, to see how period effects may have differentially affected birth cohorts
• Over successive cohorts for those age ranges captured by more than one cohort, to see how age-specific mental health has altered from cohort to cohort (cohort effects)

Due to the pooling of data and certain years being excluded due to lack of data, included age ranges were not precisely comparable between cohorts (Table 1). Preliminary analysis of narrower cohorts and/or use of datasets for individual years was piloted as a way of overcoming this, but due to small numbers it was not possible to distinguish trends from the influence of random variation. Therefore, some limitations in making direct comparisons of ages between cohorts were accepted, with this being made explicit in reporting the results. The disparity is at most one year outside the stated range for one of the two cohorts (e.g. those aged 16–32 years in the jilted generation cohort are compared with those aged 16–31 years in the generation X cohort).

**Table 1:** Age (years) of each birth cohort in all pooled datasets.

Years of data collected	Birth years of cohort				
	1916–30	1931–45	1946–60	1961–75	1976–90
	WWI	WWII	Boomers	Gen X	Jilted
1991/92	61–76	46–61	31–46	16–31	
1993/94	63–78	48–63	33–48	18–33	
1995/97	65–81	50–66	35–51	20–36	
1998/99	68–83	53–68	38–53	23–38	
2000/01	70–85	55–70	40–55	25–40	
2002/03	72–87	57–72	42–57	27–42	
2004/05	74–89	59–74	44–59	29–44	
2006/08		61–77	46–62	31–47	16–32
2009/10		64–79	49–64	34–49	19–34
2012/14		67–83	52–68	37–53	22–38

The jilted generation hypothesis was formally tested using logistic regression modelling to derive percentage change (with 95% confidence intervals) in age-specific GHQ caseness on the absolute scale between birth cohorts for each gender separately. To further investigate specific changes around the Great Recession and austerity, the same method was used to compare pre-recession, recession period and austerity period GHQ caseness for each broad age group and birth cohort by gender. As secondary analysis, models were adjusted for highest educational attainment to quantify the potential contribution of educational expansion to results.

Weights for non-response were used in all analyses. Sensitivity analysis was performed classifying those 60 years of age and over as retired to ensure any misclassification due to early retirement did not impact on results. Statistical analysis was performed in Stata SE v14, and all figures were created in Microsoft Excel (2010).

## 3 Results

### 3.1 Participants

216,068 individuals were eligible for inclusion. We excluded 15,742 individuals without recorded outcome status (7.3%), leaving 200,326 participants (89,865 males and 110,461 females) for initial analysis stratifying by broad age category. Following stratification by birth cohort 175,046 individuals remained, and the smallest group included in analysis numbered 612 individuals (Table 2).

**Table 2:** Number of participants from each birth cohort in all pooled datasets.

Years of data collected	Sex	Birth years of cohort				
		1916–30	1931–45	1946–60	1961–75	1976–90
		WWI	WWII	Boomers	Gen X	Jilted
1991/92	Men	612	685	920	867	
	Women	725	751	1024	985	
1993/94	Men	2317	3081	4053	3902	
	Women	2919	3285	4585	4512	
1995/97	Men	1661	2326	3017	2935	
	Women	2037	2629	3408	3611	
1998/99	Men	1278	2167	2782	2779	
	Women	1712	2397	3284	3408	
2000/01	Men	1281	2140	2639	2746	
	Women	1901	2359	3232	3485	
2002/03	Men	879	1859	2425	2549	
	Women	1260	2198	2942	3483	
2004/05	Men	898	1862	1495	1519	
	Women	1251	2176	1890	2013	
2006/08	Men		2441	3224	3186	2213
	Women		2786	3809	4145	2881
2009/10	Men		1039	1328	1389	963
	Women		1145	1558	1840	1306
2012/14	Men		1076	1630	1671	1196
	Women		1240	1857	2210	1777

The age distribution of respondents changed slightly over time, with the contribution of 16–30 year olds falling from 26.0% of the sample in 1991/92 to 17.9% in 2012/14, 31–64 year olds increasing from 53.2% to 57.1%, and over 65s increasing from 20.8% to 25.0% (Appendix 1). All prevalence estimates displayed and their 95% confidence intervals are included in tables as supplementary materials (Appendix 1-3).

### 3.2 Main results

#### 3.2.1 The jilted generation hypothesis

**Fig. 1:**
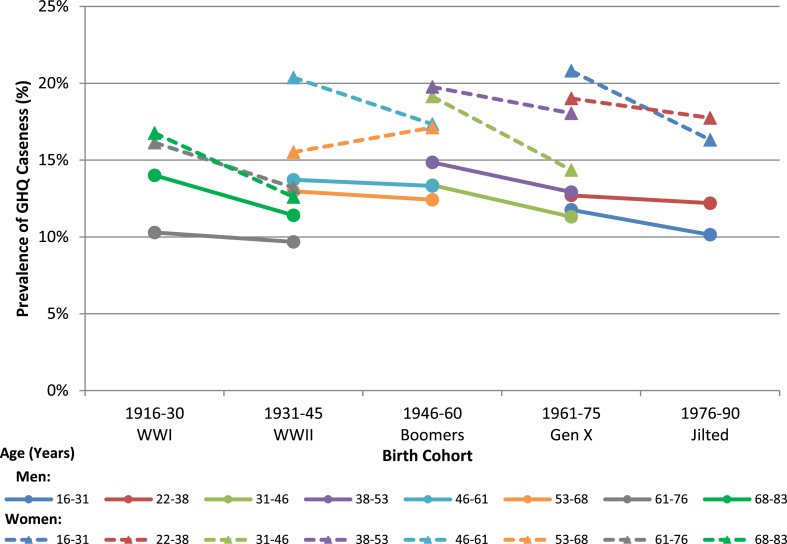
Pooled General Health Questionnaire caseness over birth cohort in males and females, 1991–2014. Dashed line indicates female birth cohorts. All estimates from two pooled years of data collection.

For all age ranges captured by more than one male birth cohort there was a sequential decrease in GHQ caseness between 0.5% (95% CI −2.9% to 1.9%) and 2.7% (−5.5%–0.18%), though these decreases did not achieve statistical significance (Fig. 1; Table 3). There was similarly an almost universal decrease in female age-specific GHQ caseness from cohort to cohort between 1.3% (95% CI −3.6% to 1.1%) and 4.4% (95% CI −7.1% to −1.5%). The decrease in GHQ caseness between female birth cohorts achieved statistical significance in five of eight comparisons, indicating an improvement in population mental health in successive birth cohorts.

**Table 3:** Percentage change in age-specific GHQ caseness (with 95% CI) between birth cohorts; p-values < 0.05 in bold.

Cohort Comparison	MEN		WOMEN	
	% Change Between Cohorts (95% CI)	p-value	% Change Between Cohorts (95% CI)	p-value
WWI to WWII (68–83 years)	−2.65% (−5.48%, 0.18%)	0.067	**−4.31% (−7.09%, −1.52%)**	**0.002**
WWI to WWII (61–76 years)	−0.61% (−3.21%, 1.99%)	0.646	**−2.79% (−5.49%, −0.09%)**	**0.043**
WWII to Boomers (53–68 years)	−0.55% (−2.78%, 1.68%)	0.627	1.59% (−0.65%, 3.83%)	0.165
WWII to Boomers (46–61 years)	−0.39% (−3.19%, 2.40%)	0.782	**−2.91% (−5.78%, −0.03%)**	**0.048**
Boomers to Gen X (38–53 years)	−1.95% (−4.17%, 0.26%)	0.084	−1.72% (−3.91%, 0.47%)	0.123
Boomers to Gen X (31–46 years)	−1.96% (−4.22%, 0.29%)	0.088	**−4.44% (−6.73%, −2.15%)**	<**0.001**
Gen X to Jilted (22–38 years)	−0.51% (−2.90%, 1.89%)	0.677	−1.28% (−3.62%, 1.06%)	0.284
Gen X to Jilted (16–31 years)	−1.56% (−3.88%, 0.76%)	0.186	**−4.25% (−6.83%, −1.66%)**	**0.001**

This between-cohort improvement for women includes the jilted generation cohort for their first recorded measurement, where they saw a 4.3% reduction in caseness compared with generation X at age 16–31 years (95% CI 1.7%–6.8%). However, there was no significant improvement in their second measurement at age 22–38 years, which coincided with the austerity period, when comparing with the previous cohort.

Secondary analysis adjusting for educational attainment explained some of the difference in age-specific GHQ caseness between cohorts for women, particularly for older cohorts where it fully explained differences between the WWI and WWI cohorts at age 61–76 years and the WWII to baby boomer cohorts at age 46–61 years (Table 4).

**Table 4:** Percentage change in age-specific GHQ caseness (with 95% CI) between birth cohorts adjusted for educational attainment; p-values < 0.05 in bold.

Cohort Comparison	MEN		WOMEN	
	% Change Between Cohorts (95% CI)	p-value	% Change Between Cohorts (95% CI)	p-value
WWI to WWII (68–83 years)	−1.77% (−4.67%, 1.14%)	0.234	**−4.28% (−7.26%, −1.31%)**	**0.005**
WWI to WWII (61–76 years)	0.49% (−2.17%, 3.15%)	0.716	−1.67% (−4.50%, 1.15%)	0.245
WWII to Boomers (53–68 years)	0.97% (−1.36%, 3.30%)	0.416	2.39% (−0.06%, 4.84%)	0.056
WWII to Boomers (46–61 years)	0.91% (−2.06%, 3.88%)	0.547	−1.78% (−4.81%, 1.25%)	0.250
Boomers to Gen X (38–53 years)	−1.41% (−3.71%, 0.89%)	0.229	−1.39% (−3.77%, 0.99%)	0.253
Boomers to Gen X (31–46 years)	−1.36% (−3.68%, 0.97%)	0.253	**−3.19% (−5.62%, −0.77%)**	**0.010**
Gen X to Jilted (22–38 years)	0.07% (−2.33%, 2.47%)	0.956	−1.10% (−3.50%, 1.31%)	0.371
Gen X to Jilted (16–31 years)	−1.52% (−3.92%, 0.87%)	0.213	**−3.23% (−5.91%, −0.54%)**	**0.019**

#### 3.2.2 The influence of age effects

**Fig. 2:**
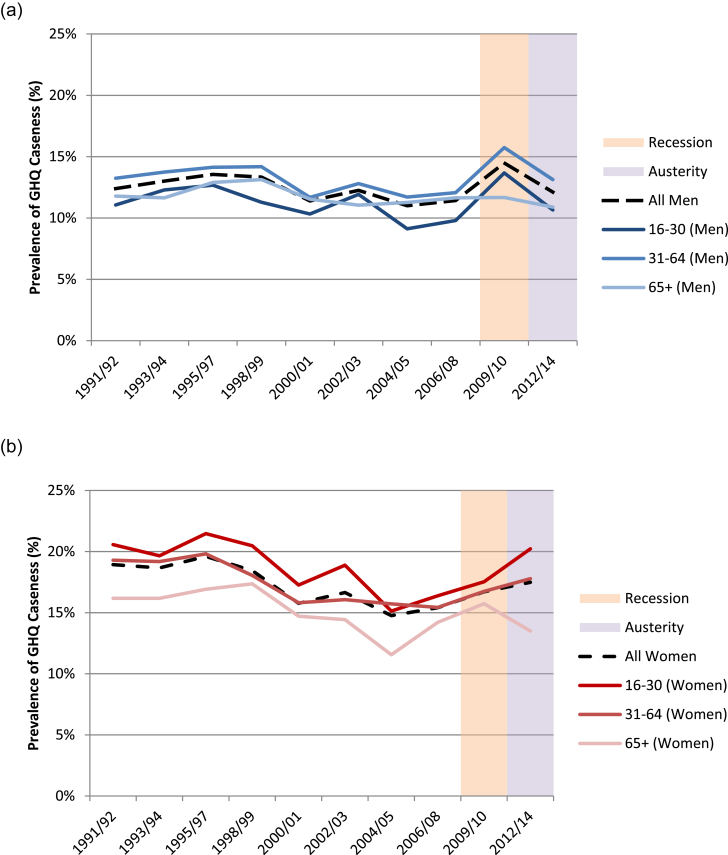
a Age-specific General Health Questionnaire caseness in males aged over 16 years, 1991–2014. Groupings: age 16–30 years (younger working-age); age 31–64 years (older working-age); age over 65 years (retirement-age). All estimates from two pooled years of data collection. b: Age-specific General Health Questionnaire caseness in females aged over 16 years, 1991–2014. Groupings: age 16–30 years (younger working-age); age 31–64 years (older working-age); age over 65 years (retirement-age). All estimates from two pooled years of data collection.

Comparing broad age groups among men (Fig. 2a), throughout most of the study period GHQ caseness was lowest for 16–30 year olds, was consistently highest for 31–64 year olds, and those over 65 years fell between. This gives the impression that male GHQ caseness may take an approximate inverted U shape, with the peak in middle age.

In contrast with age-specific GHQ caseness in men, for women caseness was almost universally at its highest in 16–30 year olds, and consistently at its lowest in those over 65, with those aged 31–64 years very close to the overall mean (Fig. 2b). This suggests mental health in women improves over the life course, with peak prevalence of poor mental health occurring during younger working-age.

Sensitivity analysis classifying older working-age as 30–59 years and retired as 60 years and above resulted in very similar trends for both genders.

**Fig. 3:**
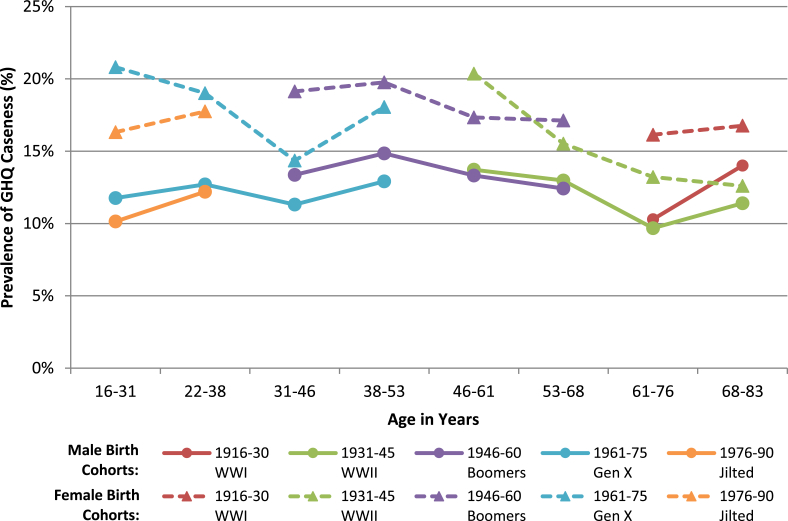
Pooled General Health Questionnaire caseness by birth cohort over life course in males and females, 1991–2014. Dashed line indicates female birth cohorts. All estimates from two pooled years of data collection.

In keeping with the age patterning seen in Fig. 2a, when considered by birth cohort GHQ caseness for men did take the form of an inverted U across most of the life course (Fig. 3). Also illustrated with the use of narrower age categories was a worsening in very old age, a finding often (though not universally) documented in the literature (Luppa et al., 2012). There was no marked impression from these results of differences in age patterning across birth cohorts for men.

Contrastingly, the pattern of GHQ caseness over the life course within female birth cohorts was less clear. Though there was an improvement in three of the five cohorts (WWII, baby boomers and generation X) comparing youngest and oldest measurement, trends were erratic, giving the impression that period and cohort effects may be contributing more than age effects alone.

#### 3.2.3 The influence of period effects including the Great Recession and austerity

##### 3.2.3.1 Period effects by broad age category

Considering period effects prior to the Great Recession (Fig. 2a and b), there was an overall trend towards improvement for all groups from 1991/92 to 2004/05 which was more marked for women, as has been reported previously (Thomson et al., 2018). There was a small increase in caseness for both genders in 2002/03, which coincided with a smaller economic downturn in 2002 (aThe Office for National Statistics, ). This potential period effect appears to have impacted only those of working-age for men, and mostly those of younger working-age for women, however the following year there was a marked reduction back to baseline for all affected groups.

During the recession period of 2009/10, the previously reported increase in caseness for men was seen (Fig. 2a). (Katikireddi et al., 2012; Thomson et al., 2018) However, while this increase affected younger and older working-age men to a similar degree, rising by 3.7% (95% CI 1.2%–6.2%) and 3.5% (95% CI 2.1%–5.0%) respectively, it was not evident in those over 65 years (Table 5). During the austerity period, both younger and older working-age men saw an almost identical recovery with a decrease of 3.0% (95% CI −6.1% to 0.1%) and 2.6% (−4.4% to −0.8%) respectively, approaching their pre-recession baseline.

**Table 5:** Percentage change in age-specific GHQ caseness (with 95% CI) between pre-recession, recession and austerity periods; p-values < 0.05 in bold.

Age Group	Sex	% Change Pre-Recession to Recession (95% CI)	p-value	% Change Recession to Austerity (95% CI)	p-value	% Change Pre-Recession to Austerity (95% CI)	p-value
16–30 Years	*Men*	**3.68% (1.20%, 6.16%)**	**0.004**	−3.02% (−6.14%, 0.10%)	0.058	0.85% (−1.40%, 3.11%)	0.456
	*Women*	1.13% (−1.60%, 3.86%)	0.417	2.71% (−0.59%, 6.00%)	0.107	**3.73% (1.25%, 6.20%)**	**0.003**
31–64 Years	*Men*	**3.51% (2.05%, 4.98%)**	<**0.001**	**−2.61% (−4.44%, −0.77%)**	**0.005**	1.05% (−0.34%, 2.44%)	0.138
	*Women*	1.29% (−0.14%, 2.73%)	0.078	1.04% (−0.66%, 2.74%)	0.229	**2.30% (0.98%, 3.61%)**	**0.001**
65+ Years	*Men*	0.03% (−2.17%, 2.23%)	0.979	−0.78% (−3.18%, 1.63%)	0.527	−0.76% (−2.76%, 1.25%)	0.460
	*Women*	1.49% (−0.66%, 3.65%)	0.174	−2.23% (−4.68%, 0.22%)	0.075	−0.72% (−2.71%, 1.28%)	0.481

A similar degree of non-significant increased caseness was seen during the recession period for each female age group, ranging from 1.1% (95% CI −1.6% to 3.9%) for younger working-age to 1.5% (95% CI −0.7% to 3.7%) for retirement-age women compared with pre-recession (Fig. 2b; Table 5). However, in contrast with men, during the austerity period each age group experienced differing trajectories. For those over 65 there was a 2.2% decrease in caseness (95% CI −4.7% to 0.2%); for those aged 31–64 years there was a 1.0% increase in caseness (95% CI −0.7% to 2.7%); and for those aged 16–30 years there was a larger increase of 2.7% (95% CI −0.6% to 6.0%). While these sequential increases were not statistically significant when considered alone, measuring the whole period from pre-recession to austerity there is a clear and significant increase in caseness for both younger and older working-age women of 3.7% (95% CI 1.3%–6.2%) and 2.3% (95% CI 1.0%–3.6%) respectively, while men see recovery back to pre-recession baseline.

##### 3.2.3.2 Period effects by birth cohort

**Fig. 4:**
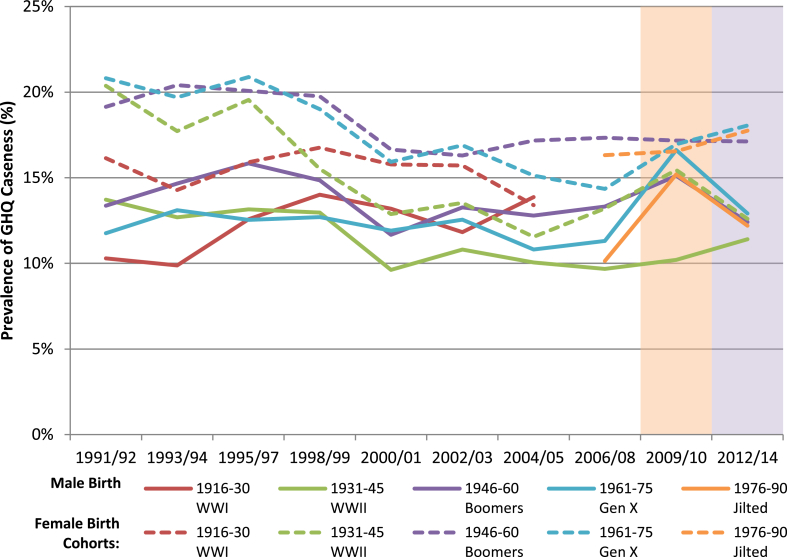
Pooled General Health Questionnaire caseness by birth cohort over time in males and females, 1991–2014. Dashed line indicates female birth cohorts. All estimates from two pooled years of data collection.

Considering period effects prior to the Great Recession by birth cohort, the trajectories of both war cohorts and the generation X cohort appear relatively in keeping with expected age effects (Fig. 4). The smaller period effect in 2002/03 resulted in a small increase in GHQ caseness in all except the oldest WWI cohort, who appear to have been relatively protected from this. Immediately following this there was good recovery for most affected groups, with the exception of the two baby boomer cohorts. From 2004/05 (when aged 44–59 years) the male baby boomer cohort did not see the anticipated recovery and improvement of late middle/early older age, with trends remaining flat up to the Great Recession. The female baby boomer cohort followed a similar trajectory, seeming to follow their expected life course trajectory of reducing caseness until 2002/03 when their trend also became static with none of the expected further improvement with age, such that both the generation X and jilted generation cohorts had better mental health throughout much of the 2000s despite being 15–30 years younger.

Considering the period around the Great Recession and subsequent onset of austerity, the youngest two birth cohorts see the greatest percentage change in caseness (Table 6). The male generation X cohort experienced a 5.0% increase (95% CI 2.9%–7.1%) immediately following the recession, but see a significant 3.7% reduction (95% CI −6.4% to −1.0%) following the onset of austerity. The female generation X cohort also experiences a smaller increase immediately following the recession of 2.5% (95% CI 0.5%–4.6%) but has no significant improvement during austerity; in fact there is a non-significant further increase of 1.1% (95% CI −1.4% to 3.6%). The male jilted cohort also experienced an increase in caseness following the recession of 4.8% (95% CI 2.2%–7.3%), followed by a reduction of 3.0% during austerity (95% CI −6.3% to 0.3%). Their female counterparts experienced little change immediately following the recession but a small increase of 1.2% (95% CI −1.7% to 4.2%) following the onset of austerity, though this did not reach statistical significance. Comparing the whole time period, only the female generation X cohort experienced a statistically significant increase in caseness from pre-recession to the austerity period of 3.6% (95% CI 1.7%–5.4%).

**Table 6:** Percentage change in GHQ caseness (with 95% CI) for each birth cohort between pre-recession, recession and austerity periods; p-values < 0.05 in bold.

Birth Cohort	Sex	% Change Pre-Recession to Recession (95% CI)	p-value	% Change Recession to Austerity (95% CI)	p-value	% Change Pre-Recession to Austerity (95% CI)	p-value
WWII	*Men*	0.52% (−1.70%, 2.74%)	0.647	1.21% (−1.51%, 3.93%)	0.385	1.68% (−0.45%, 3.80%)	0.122
	*Women*	2.18% (−0.21%, 4.57%)	0.074	−2.87% (−5.80%, 0.06%)	0.055	−0.61% (−2.94%, 1.72%)	0.606
Boomers	*Men*	1.75% (−0.52%, 4.01%)	0.131	**−2.70% (−5.37%, −0.03%)**	**0.047**	−0.92% (−3.07%, 1.23%)	0.401
	*Women*	−0.17% (−2.51%, 2.17%)	0.886	−0.04% (−2.70%, 2.61%)	0.974	−0.22% (−2.37%, 1.93%)	0.844
Gen X	*Men*	**4.99% (2.85%, 7.12%)**	<**0.001**	**−3.71% (−6.44%, −0.98%)**	**0.008**	1.57% (−0.39%, 3.53%)	0.117
	*Women*	**2.54% (0.52%, 4.56%)**	**0.014**	1.09% (−1.39%, 3.57%)	0.388	**3.57% (1.71%, 5.42%)**	<**0.001**
Jilted	*Men*	**4.75% (2.22%, 7.28%)**	<**0.001**	−3.01% (−6.26%, 0.25%)	0.070	2.00% (−0.28%, 4.27%)	0.085
	*Women*	0.23% (−2.41%, 2.87%)	0.864	1.20% (−1.74%, 4.15%)	0.424	1.41% (−0.87%, 3.70%)	0.226

In contrast with between-cohort regression models, secondary analysis considering educational attainment as a covariate had no marked influence when examining changes for groups around the recession and austerity periods.

## 4 Discussion

### 4.1 Statement of principal findings

We found no evidence to support the jilted generation hypothesis in relation to mental health, and in fact found evidence of a consistent statistically significant improvement in mental health from cohort to cohort over time for women. We also found that, while the previously reported worsening of male mental health which occurred immediately following the recession (4) was experienced evenly across working-age groups, for women the worsening of mental health following the onset of austerity (9) was disproportionately experienced by women of younger working-age. Finally, the mental health of the retirement-age population of both genders did not alter markedly during either the recession or austerity periods. While it is not possible to draw definitive causal conclusions from this study, our findings are useful in examining changes in secular trends and their chronological association with macroeconomic events and policies.

### 4.2 Strengths and limitations

Our study has important strengths which make its findings relevant and of public health importance. The HSE is a large, nationally and regionally representative survey (NatCen Social Research and UK Data Service, 1991–2014), and the long time period over which it has run annually with the same methodology allows for consideration of population mental health outcomes in the context of very long-term trends, using a validated outcome measurement tool. The decision to present age-period-cohort data descriptively eliminates the need to make the highly subjective assumptions required to perform age-period-cohort statistical modelling techniques (Bell, 2014), described as being based on intuition (Bell and Jones, 2014), allowing the reader to interpret trends independently with less influence from the researcher.

This study also has some limitations which should be considered in interpreting its findings. The use of repeat cross-sectional rather than longitudinal data limits the ability to draw causal inferences, as is acknowledged throughout. It is also challenging with annually collected data to determine definitively when macroeconomic events or austerity measures may have begun to impact on health for individuals sampled. It is unfortunate that due to the administration of the HSE, data were not available from 2007, 2011, 2013 or 2015, as this would have strengthened the evidence for assessment of trends. Stratification by sex and age group or birth cohort reduced sample size, increasing imprecision around population estimates. This was overcome by pooling data from two years for analyses, meaning no individual group had fewer than 612 individuals and only 10.7% of groups had less than 1000 participants – 73.3% of these were in 1991/92, outwith the main time period of interest (Table 2). However, pooling of data and missing years meant age-specific estimates were not directly comparable between cohorts, which is acknowledged throughout. As birth cohorts spanned large age ranges to avoid small, unrepresentative samples, age-specific caseness could only ever be compared between two birth cohorts. However, the fact that the finding of improvement in mental health between cohorts was consistent across pairings does make this conclusion more justified. Survey non-response could potentially bias our assessment of trends, particularly since response rates have declined over time (Gorman et al., 2014). We have attempted to limit this bias by applying survey weights in all analyses.

### 4.3 Our findings in context

#### 4.3.1 Age effects

Our study found that age-specific population mental health in men has followed similar patterns to the trends for all men throughout the study period, with the exception of periods which coincide with economic downturn when those over 65 years do not experience marked change while other groups experience worsening. Given the relative protection of the retired population from unemployment and welfare reforms in the United Kingdom, this adds some weight to the association potentially being causal (bThe Office for National Statistics, ; Copeland et al., 2015). It is noted that this protection of the older population from material disadvantage following the recession has not been the case in all international settings (Stuckler and Basu, 2013), and similar work in these settings finding that older men not financially protected experienced worsening mental health would be needed to add strength to this argument. Over the life course, mental health appears best in younger working-age men, worsening in middle age, and falling again at retirement-age. Our finding of a mid-life ‘hump’ in poor mental health is in keeping with much of the existing literature around prevalence of poor mental health over the life course (Blanchflower and Oswald, 2008). Some have suggested that the hump may be more exaggerated in those of lower socioeconomic position (Lang et al., 2011), but we were unable to test this hypothesis due to the reduction in sample size associated with further stratification of groups.

Our study suggests male mental health may worsen in very old age. The existing literature around this is conflicting, with some finding mental health in over 75s is among the best for both genders with no evidence of worsening (McManus et al., 2016), and some reporting a steady deterioration secondary to rising isolation and dementia prevalence (Bell, 2014; Luppa et al., 2012). It has been suggested that, due to the complex presentation of their mental health problems, presence of physical co-morbidities, and heterogeneity of the older population, analysis using generic screening tools rather than symptom rating scales for the elderly may be insufficient for this subset of the population (Luppa et al., 2012).

For women, our study found age-specific poor mental health in each group approximated the average trend for all women prior to 2010, as was seen for males, with similar relative protection of those of retirement-age from period effects. Over the life course, female mental health appears to be consistently poorest in younger working-age women, improves in older working-age, and is at its best over the age of 65 years, with no evidence of a worsening in old age as seen in men. This is at odds with existing literature, much of which does not find a difference between male and female trends across the life course (Bell, 2014; Blanchflower and Oswald, 2008; Lang et al., 2011), and with those that do largely attributing it to a period or cohort effect at that particular time such as austerity (McManus et al., 2016). However Fig. 2b illustrates that, in this population of women, the patterning of poor mental health by age group over the life course has been consistent throughout the study period. Further differentiation into smaller age bands may be useful to see whether the trend of continuous improvement remains as robust.

#### 4.3.2 Period effects

For both genders there appear to be period effects on population mental health for those of working-age beginning in 2002 and 2009, which coincide with economic downturns. The shorter period effect in 2002 most affected those in the baby boomer cohort (born 1946–1960) with a possible lag effect lasting until after the 2008 recession, particularly for women, which has resulted in this cohort not seeing their anticipated improvement in mental health with increasing age. This mirrors findings from another 2014 UK study where a developing cohort effect was described in this age group, though as with their results inference should be cautious as sample sizes in these stratified groups are relatively small (McManus et al., 2016). Regardless, the finding being replicated in two samples adds weight.

Contrastingly, the period effect which coincided with the 2008 recession appears to have most affected younger birth cohorts in men, impacting similarly on those born between 1961 and 1990. The relative improvement in population mental health seen for men throughout the subsequent austerity period was experienced to a similar degree by all birth cohorts, with no one cohort seeing long-term or disproportionate disadvantage. As has been discussed in the literature (Thomson et al., 2018), it remains unclear why male mental health in England appears to have recovered so quickly following the Great Recession despite the extreme initial deterioration. Though this is not entirely out of keeping with the sharp, short-term deteriorations often seen for men following historic macroeconomic shocks (Chang et al., 2009), the lack of longitudinal studies has made it difficult to determine a clear pattern to these longer-term consequences (Frasquilho et al., 2016).

It appears the worsening of female mental health which coincided with the post-2010 austerity period may have been disproportionately experienced by women of working-age, particularly women of younger working-age. This is in keeping with recent work highlighting this group as particularly high-risk for poor mental health (McManus et al., 2016; Government Social Research, 2017; Knudsen et al., 2016; Lessof et al., 2016). It also adds weight to the hypothesis that welfare reforms may be causally implicated in these changes, as working-age women (particularly those younger and supporting families) have been disproportionately impacted, shouldering 85% of the financial losses (Ariss et al., 2015). It would be useful to examine more recent trends to determine whether the differing trajectories for women of younger and older working-age which appear to be becoming established here continued with the further expansion of more severe austerity policies from 2015 onwards (Beatty and Fothergill, 2016).

#### 4.3.3 Cohort effects

Our study found that, over time, population mental health is improving from cohort to cohort, and that this trend holds true despite the potential period effects described above. This finding of a consistent cohort improvement in mental health is at odds with the most recent work considering the UK population. Using longitudinal data from the British Household Panel Study and its successor Understanding Society between 1991 and 2008, Bell et al. found that more recent cohorts have worse mental health (Bell, 2014). In contrast, Spiers et al. reported no evidence of significant cohort effects in poor mental health from 1993 to 2007 using data from three cross-sectional National Psychiatric Morbidity Surveys (Spiers et al., 2011), while Rice et al. found the highest prevalence of poor mental health for the 'baby boomer' cohort using Health Survey for England data from 1994 to 2007 (Rice et al., 2010). However, in all of these analyses one of either age or period effects were constrained to be zero, based on the assumptions of the researchers. Given the historic literature on the impact of economic crises and age on mental health (Frasquilho et al., 2016; Blanchflower and Oswald, 2008), and our findings, it could be argued that this assumption was not justified.

We found that between-cohort improvements in mental health have been much more marked in women than in men. This is in keeping with findings from our previous work (Thomson et al., 2018), which reported a greater degree of overall improvement in population mental health for women than men during the same time period. There are some interesting hypotheses to be explored here around whether the changing role of women in society throughout the 20th century may have played a part in this trend towards improvement; for example, through increasing access to education, participation in the workforce, social independence and control of reproductive rights (Zweiniger-Bargielowska, 2014). This would be in keeping with our secondary analysis which found that educational expansion may play a role in explaining some of the between-cohort improvement. Potentially supporting this interpretation, a large cross-national survey by the World Health Organisation in 2009 also found an apparent cohort effect in women, with lower levels of depression in younger cohorts, which appeared to be related to changes in female gender roles (Seedat et al., 2009).

The period effects on population mental health discussed throughout this paper which coincide with the Great Recession and subsequent austerity period appear in both genders to have been most felt by the two youngest considered birth cohorts born in 1961-75 and 1976-90. Of concern, for women there has not yet been a clear improvement for these younger working-age groups, with a continued worsening of mental health to the point that a true cohort effect may be being established where these birth cohorts continue to have poorer mental health than expected for their age.

### 4.4 Conclusion and implications for policymakers

Overall, mental health in England has consistently improved from generation to generation, particularly among women, which likely at least in part reflects positive changes in social, economic, and mental health policy in the last century. However it appears that, following the 2008 recession and subsequent austerity measures, this pattern may be altering for younger generations of women, while those of retirement-age of both genders have been relatively well protected.

These findings imply that economic policy decisions in the aftermath of the recession, which are still actively being pursued (BBC News, 2018), could be disproportionately and negatively impacting the mental health of a jilted generation who have already been materially disadvantaged by policy decisions in the past (Howker, 2013). While further longitudinal studies are needed to demonstrate a definitive causal link, given these findings in the context of a wider growing evidence base linking austerity policies to other negative health consequences and health inequalities (Stuckler et al., 2017), policymakers should strongly consider whether such policies are in the best interest of the whole population.

## Competing interests

The authors declare no conflicts of interest.
