# Supplementary material for: Mental health and the jilted generation: Using age-period-cohort analysis to assess differential trends in young people's mental health following the Great Recession and austerity in England
Source: Soc Sci Med. 2018 Oct;214:133–43. doi: 10.1016/j.socscimed.2018.08.034 (PMC6176124; doi:10.1016/j.socscimed.2018.08.034)
Supplement: Multimedia component 1 [file mmc1.docx]

**APPENDIX**

| Appendix 1: Pooled age-specific GHQ caseness (with 95% CI) in over 16s by gender, 1991-2014 | | | | | | | | | |
| --- | --- | --- | --- | --- | --- | --- | --- | --- | --- |
| Pooled Years | **Sex** | **All (%)** | ***n*** | **16-30 (%)** | ***n*** | **31-64 (%)** | ***n*** | **65+ (%)** | ***n*** |
| 1991/  1992 | *Men* | 12.39  (11.27-13.51) | *3300* | 11.07  (8.98-13.16) | *867* | 13.24  (11.68-14.81) | *1797* | 11.79  (9.29-14.30) | *636* |
|  | *Women* | 18.93  (17.69-20.18) | *3824* | 20.57  (18.05-23.09) | *987* | 19.30  (18.05-23.09) | *1990* | 16.17  (13.69-18.66) | *847* |
| 1993/  1994 | *Men* | 13.00  (12.46-13.55) | *14387* | 12.29  (11.23-13.35) | *3693* | 13.75  (13.00-14.50) | *8160* | 11.64  (10.39-12.89) | *2534* |
|  | *Women* | 18.66  (18.07-19.25) | *16831* | 19.66  (18.45-20.86) | *4192* | 19.19  (18.38-20.00) | *9048* | 16.18  (14.97-17.38) | *3591* |
| 1995/  1997 | *Men* | 13.56  (12.91-14.20) | *10932* | 12.67  (11.38-13.96) | *2557* | 14.13  (13.27-14.99) | *6320* | 12.90  (11.45-14.34) | *2055* |
|  | *Women* | 19.60  (18.91-20.28) | *13059* | 21.47  (20.03-22.92) | *3111* | 19.81  (18.89-20.73) | *7198* | 16.91  (15.51-18.31) | *2750* |
| 1998/  1999 | *Men* | 13.34  (12.68-14.00) | *10191* | 11.28  (9.98-12.58) | *2270* | 14.18  (13.30-15.07) | *5979* | 13.13  (11.63-14.63) | *1942* |
|  | *Women* | 18.44  (17.75-19.12) | *12306* | 20.47  (18.95-22.00) | *2701* | 18.05  (17.15-18.94) | *7076* | 17.36  (15.88-18.83) | *2529* |
| 2000/  2001 | *Men* | 11.39  (10.73-12.05) | *10299* | 10.33  (9.02-11.64) | *2072* | 11.68  (10.87-12.50) | *5923* | 11.54  (9.99-13.09) | *2304* |
|  | *Women* | 15.76  (15.09-16.43) | *13043* | 17.26  (15.77-18.75) | *2474* | 15.81  (14.97-16.65) | *7211* | 14.71  (13.23-16.18) | *3358* |
| 2002/  2003 | *Men* | 12.26  (11.61-12.90) | *10342* | 11.92  (10.76-13.09) | *3031* | 12.80  (11.90-13.70) | *5525* | 11.04  (9.58-12.50) | *1786* |
|  | *Women* | 16.65  (16.00-17.29) | *13103* | 18.88  (17.61-20.14) | *3719* | 16.06  (15.19-16.93) | *6986* | 14.43  (13.02-15.85) | *2398* |
| 2004/  2005 | *Men* | 11.00  (10.17-11.85) | *6767* | 9.12  (7.38-10.86) | *1082* | 11.70  (10.59-12.82) | *3417* | 11.26  (9.41-13.10) | *2268* |
|  | *Women* | 14.76  (13.91-15.60) | *8626* | 15.13  (13.17-17.10) | *1367* | 15.72  (14.60-16.83) | *4402* | 11.56  (9.90-13.22) | *2857* |
| 2006/  2008 | *Men* | 11.43  (10.84-12.02) | *12123* | 9.79  (8.51-11.08) | *2248* | 12.07  (11.29-12.85) | *7128* | 11.64  (10.43-12.85) | *2747* |
|  | *Women* | 15.40  (14.81-16.00) | *15124* | 16.38  (14.98-17.79) | *2852* | 15.43  (14.67-16.20) | *8859* | 14.21  (13.03-15.39) | *3413* |
| 2009/  2010 | *Men* | 14.48  (13.43-15.54) | *5258* | 13.67  (11.25-16.09) | *948* | 15.74  (14.35-17.13) | *3017* | 11.67  (9.83-13.51) | *1293* |
|  | *Women* | 16.71  (15.73-17.69) | *6590* | 17.53  (15.12-19.94) | *1204* | 16.74  (15.48-18.00) | *3827* | 15.73  (13.84-17.61) | *1559* |
| 2012/  2014 | *Men* | 12.11  (11.23-12.99) | *6266* | 10.66  (8.73-12.59) | *1092* | 13.13  (11.94-14.32) | *3551* | 10.89  (9.34-12.44) | *1623* |
|  | *Women* | 17.48  (16.60-18.37) | *7955* | 20.23  (18.00-22.46) | *1449* | 17.78  (16.64-18.92) | *4575* | 13.50  (11.94-15.06) | *1931* |

| Appendix 2: Pooled GHQ caseness by birth cohort in men over 16 years, 1991-2014 | | | | | | |
| --- | --- | --- | --- | --- | --- | --- |
| Year | **All (%)** | **1916-30 (%)** | **1931-45 (%)** | **1946-60 (%)** | **1961-75 (%)** | **1976-90 (%)** |
| 1991/  1992 | 12.39  (11.22-13.55) | 10.29  (7.89-12.70) | 13.72  (11.15-16.30) | 13.37  (11.17-15.57) | 11.76  (9.62-13.91) |  |
| *n* | *3084* | *612* | *685* | *920* | *867* |  |
| 1993/  1994 | 12.92  (12.35-13.49) | 9.88  (8.67-11.10) | 12.69  (11.52-13.87) | 14.66  (13.47-15.74) | 13.10  (12.04-14.15) |  |
| *n* | *13353* | *2317* | *3081* | *4053* | *3902* |  |
| 1995/  1997 | 13.69  (13.02-14.37) | 12.58  (10.99-14.18) | 13.16  (11.78-14.53) | 15.84  (14.54-17.15) | 12.54  (11.34-13.74) |  |
| *n* | *9939* | *1661* | *2326* | *3017* | *2935* |  |
| 1998/  1999 | 13.61  (12.90-14.32) | 14.01  (12.10-15.91) | 12.97  (11.55-14.38) | 14.85  (13.52-16.17) | 12.70  (11.46-13.94) |  |
| *n* | *9006* | *1278* | *2167* | *2782* | *2779* |  |
| 2000/  2001 | 11.48  (10.77-12.20) | 13.19  (10.98-15.40) | 9.62  (8.32-10.93) | 11.67  (10.45-12.90) | 11.91  (10.70-13.12) |  |
| *n* | *8806* | *1281* | *2140* | *2639* | *2746* |  |
| 2002/  2003 | 12.32  (11.57-13.07) | 11.83  (9.69-13.97) | 10.81  (9.38-12.24) | 13.27  (11.90-14.64) | 12.55  (11.24-13.86) |  |
| *n* | *7712* | *879* | *1859* | *2425* | *2549* |  |
| 2004/  2005 | 11.54  (10.59-12.48) | 13.87  (10.70-17.05) | 10.05  (8.31-11.78) | 12.79  (11.04-14.53) | 10.81  (9.21-12.42) |  |
| *n* | *5774* | *898* | *1862* | *1495* | *1519* |  |
| 2006/  2008 | 11.27  (10.65-11.88) |  | 9.68  (8.49-10.86) | 13.32  (12.11-14.53) | 11.31  (10.18-12.44) | 10.14  (8.82-11.45) |
| *n* | *11064* |  | *2441* | *3224* | *3186* | *2213* |
| 2009/  2010 | 14.80  (13.67-15.93) |  | 10.20  (8.27-12.13) | 15.11  (13.08-17.14) | 16.62  (14.51-18.73) | 15.20  (12.67-17.72) |
| *n* | *4719* |  | *1039* | *1328* | *1389* | *963* |
| 2012/  2014 | 12.37  (11.42-13.32) |  | 11.41  (9.47-13.35) | 12.42  (10.71-14.12) | 12.92  (11.20-14.63) | 12.20  (10.17-14.22) |
| *n* | *5573* |  | *1076* | *1630* | *1671* | *1196* |

| Appendix 3: Pooled GHQ caseness by birth cohort in women over 16 years, 1991-2014 | | | | | | |
| --- | --- | --- | --- | --- | --- | --- |
| Year | **All (%)** | **1916-30 (%)** | **1931-45 (%)** | **1946-60 (%)** | **1961-75 (%)** | **1976-90 (%)** |
| 1991/  1992 | 19.25  (17.94-20.56) | 16.14  (14.46-18.82) | 20.37  (17.49-23.25) | 19.14  (16.73-21.55) | 20.81  (18.28-23.35) |  |
| *n* | *3485* | *725* | *751* | *1024* | *985* |  |
| 1993/  1994 | 18.46  (17.84-19.07) | 14.29  (13.02-15.56) | 17.72  (16.41-19.02) | 20.41  (19.25-21.58) | 19.70  (18.54-20.86) |  |
| *n* | *15301* | *2919* | *3285* | *4585* | *4512* |  |
| 1995/  1997 | 19.48  (18.76-20.20) | 15.91  (14.32-17.49) | 19.55  (18.04-21.07) | 20.07  (18.73-21.42) | 20.88  (19.55-22.21) |  |
| *n* | *11685* | *2037* | *2629* | *3408* | *3611* |  |
| 1998/  1999 | 18.11  (17.38-18.84) | 16.76  (14.99-18.53) | 15.52  (14.07-16.97) | 19.76  (18.40-21.12) | 19.01  (17.70-20.33) |  |
| *n* | *10801* | *1712* | *2397* | *3284* | *3408* |  |
| 2000/  2001 | 15.40  (14.68-16.12) | 15.78  (13.83-17.74) | 12.88  (11.44-14.32) | 16.64  (15.36-17.93) | 15.93  (14.71-17.14) |  |
| *n* | *10977* | *1901* | *2359* | *3232* | *3485* |  |
| 2002/  2003 | 15.85  (15.13-16.58) | 15.71  (13.68-17.73) | 13.53  (12.09-14.98) | 16.30  (14.95-17.64) | 16.90  (15.65-18.16) |  |
| *n* | *9883* | *1260* | *2198* | *2942* | *3483* |  |
| 2004/  2005 | 14.74  (13.81-15.67) | 13.40  (10.70-16.11) | 11.55  (9.90-13.20) | 17.17  (15.40-18.93) | 15.12  (13.50-16.74) |  |
| *n* | *7330* | *1251* | *2176* | *1890* | *2013* |  |
| 2006/  2008 | 15.42  (14.80-16.05) |  | 13.21  (11.93-14.48) | 17.34  (16.12-18.56) | 14.35  (13.27-15.44) | 16.32  (14.92-17.71) |
| *n* | *13621* |  | *2786* | *3809* | *4145* | *2881* |
| 2009/  2010 | 16.65  (15.61-17.68) |  | 15.45  (13.27-17.63) | 17.17  (15.18-19.16) | 16.96  (15.14-18.79) | 16.55  (14.29-18.80) |
| *n* | *5849* |  | *1145* | *1558* | *1840* | *1306* |
| 2012/  2014 | 16.90  (15.99-17.82) |  | 12.60  (10.71-14.50) | 17.12  (15.36-18.88) | 18.05  (16.39-19.72) | 17.75  (15.86-19.63) |
| *n* | *7084* |  | *1240* | *1857* | *2210* | *1777* |
